# Editorial: Mental health equity

**DOI:** 10.3389/fpsyt.2023.1303277

**Published:** 2023-10-31

**Authors:** April Joy Damian, Benjamin F. Miller, Joseph J. Gallo

**Affiliations:** ^1^Weitzman Institute, Washington, DC, United States; ^2^Department of Mental Health, Johns Hopkins Bloomberg School of Public Health, Baltimore, MD, United States; ^3^Department of Psychiatry and Behavioral Sciences, School of Medicine, Stanford University, Stanford, CA, United States

**Keywords:** health equity (MeSH), mental health, healthcare delivery systems, workforce, health justice

This Research Topic aims to move beyond a synthesis of what is already known about mental health in the context of health equity, and instead showcase transformative solutions, recommendations, and research applications that have real-world implications on policy, practice, and future scholarship. Furthermore, the papers included in this Research Topic elevate the importance of transdisciplinary research, draw from various methodologies that highlight lived experiences, and apply a more critical lens at how structures of power and privilege have contributed to present-day inequities, particularly those seen in mental healthcare access, utilization, and outcomes.

A foundational aspect of this work is our delivery system. The following articles tackled delivery reform in three very different ways:

McKune et al. performed a longitudinal study that examined risk factors associated with symptoms of anxiety, depression, and obsessive-compulsive disorder (OCD) among K-12 public school children in Florida during the first year of the COVID-19 pandemic. The authors found that the prevalence of anxiety, depression, or OCD fluctuated from 47.1% (April 2020) to 57.2% (October 2020) to 42.2% (April 2021). Those at highest risk for mental health issues were BIPOC (Black, Indigenous, People of Color) children, students who had lost a family member from COVID-19, and those who were identified as at-risk in previous assessments. The study underscores the need for targeted mental health interventions and screenings, particularly among BIPOC children, during situations like COVID-19.

Toro-Devia and Leyton performed a secondary analysis of data from public and private insurance sources to examine the implications of COVID-19 on the utilization of Chile's Guaranteed Explicit Health Benefits Plan (GES) for mental health. Surprisingly, during the pandemic, mental illness cases decreased by 10.5% in public insurance and 28.7% in private insurance. Conversely, psychiatric consultations outside of the GES plan doubled in 2020 in private insurance. These findings suggest that while the demand for mental healthcare rose during the pandemic, both public and private health insurance systems witnessed a decrease in admissions to the GES universal plan. The study underscores potential weaknesses in a universal guaranteed plan within an individual contribution system.

Mallonee et al. conducted semi-structured focus groups to understand the challenges faced by Hispanic adults in accessing professional mental health treatment in the Paso del Norte U.S.-Mexico border region. The qualitative analysis revealed several key themes, including participants' understanding of how to get help, and what they think mental health agencies, providers, and researchers should do to improve access. These findings also underscore the pressing need for innovative approaches to mental health engagement in the region and highlight the importance of tailoring mental health initiatives to the specific needs and cultural contexts of Hispanic communities in the border region.

This is followed by a set of articles highlighting *Mental health equity* as it relates to the workforce:

Chen and Wang used national data from the Health Survey for England to study the prevalence of common mental health problems according to industrial classification from 2014 to 2018. The highest rate of common mental health problems was found in the unemployed with a third scoring above the threshold on the General Health Questionnaire (GHQ-12). Comparing industries over time revealed wide variation in rates, but a common trend was an increasing disparity in the rate of distress within industries between men and women.

Fu et al. studied Chinese nurses to delineate the association between social support and depressive symptoms among nurses with formal vs. contract employment. Compared to population norms, Chinese nurses in the study had a significantly higher prevalence of depression symptoms and lower social support. Nurses with less social support had higher depressive symptoms, more so among nurses with contract employment. Findings from the study suggest nurses in contract jobs have more job insecurity for which objective and subjective social support may act as a buffer against depression.

Ghebrehiwet et al. described the development of a postgraduate psychiatry training program in Liberia to address the critical shortage of trained psychiatrists and other mental health workers. The publication outlines steps taken to inaugurate a new psychiatry training program built on the foundation of a longstanding cross-country academic affiliation and national policy encouraging local capacity building. Significant challenges had to be overcome: stakeholder engagement at the national level; recruiting residency candidates; and, balancing the clinical work of the only two in-country psychiatrists with curriculum development, teaching, and advocacy.

We close out this Research Topic with a set of insightful articles presenting innovative frameworks in *Mental health equity* research:

Alemu et al. present an overview of the history of how Western-derived psychiatry and the over-reliance on Western-derived taxonomies are linked to the ongoing present-day challenges with untreated mental health challenges in many African countries. Decolonizing mental healthcare through the network approach is proposed as a viable means for addressing global mental health inequity by alleviating stigma toward mental health problems, and encouraging local researchers to pioneer context-based knowledge production and treatment design.

Faber et al. critically examine the experiences of Black people living in the United States and Canada with early schizophrenia-spectrum disorders. Racial differences in the diagnosis of schizophrenia-spectrum disorders, including misdiagnoses and barriers to treatment for Black communities, compounded by implicit bias and institutional racism in both medicine and law enforcement, are also explored. Improving diagnostic accuracy and implementing anti-racism training for mental health clinicians are among the host of recommendations proposed.

Adams and Thorpe highlight existing knowledge gaps in understanding suicide prevention for Black men and boys and offer a set of key recommendations including (1) Prioritizing frameworks centered on Black male suicide prevention, (2) Addressing innovative solutions to maintain continuity of care for Black males, (3) Enhancing research approaches to better capture the heterogeneity of Black males in suicide research, (4) Leveraging advancements in crisis support hotlines and safety planning to better serve Black males, and (5) Placing community stakeholders at the forefront of solution-driven suicide prevention research.

The Guest Editors would like to thank all the authors and reviewers for their work and devotion to the Research Topic, and hope that it can inspire further research in *Mental health equity*.

## Author contributions

AD: Writing – original draft, Writing – review & editing. BM: Writing – original draft. JG: Writing – original draft.

